# Fetal alcohol spectrum disorders prevention and clinical guidelines research-*workshop report*

**DOI:** 10.1186/s12919-023-00272-z

**Published:** 2023-08-15

**Authors:** Tracey Pérez Koehlmoos, Elizabeth Lee, Jennifer Wisdahl, Tom Donaldson

**Affiliations:** 1 Center for Health Services Research, Uniformed Services University of the Health Sciences, 4301 Jones Bridge Road, Bethesda, MD 20814 USA; 2 Department of Pediatrics, Division of Pediatric Health Systems Research and Clinical Epidemiology, Uniformed Services University of the Health Sciences, 4301 Jones Bridge Road, Building 61 Room E225, Bethesda, MD 20814 USA; 3 FASD United, 1200 Eton Ct NW, 3rd Floor, Washington, DC 20007 USA

## Abstract

It is estimated that up to 1 in 20 people in the United States are affected by fetal alcohol spectrum disorders (FASD), an array of cognitive, emotional, physical and social disorders caused by exposure to alcohol during prenatal development. Common diagnoses encompassed within FASD include mood and behavioral disorders, memory and central nervous system deficits, attention-deficit/hyperactivity disorder (ADHD), slow growth and low body weight. While this condition affects a broad range of individuals and families, it is of particular concern in the military community, where cultural factors including an increased prevalence of alcohol misuse pose a unique set of challenges. To shed light on these issues and provide an overview of the existing research, programs, and clinical practice guidelines surrounding FASD, the Uniformed Services University of the Health Sciences (USUHS), in conjunction with FASD United, hosted the *Workshop on Fetal Alcohol Spectrum Disorders Prevention and Clinical Guidelines Research* on 21 September 2022 in Washington, DC. More than 50 attendees from academia, healthcare, federal agencies, and consumer advocacy organizations gathered to share research findings, lived experiences, and strategies for improving FASD prevention, diagnosis, interventions, and support.

The workshop began with a series of presentations on FASD risk factors and causes, strategies for diagnosis and interventions, and impacts and lived experiences. Individuals and families affected by FASD spoke about the ways FASD, its symptoms, and the social stigma associated with it influences their daily lives, experiences at school and work, and access to healthcare. Several speakers highlighted the work of non-profit organizations and advocacy groups in supporting families affected by FASD and other challenges faced by military families more broadly. The workshop closed with a discussion of federal agency perspectives highlighting initiatives aimed at advancing research and access to care for women and families at-risk and those currently affected by FASD.

## Background

The Center for Health Services Research (CHSR) at USUHS is partnering with FASD United and the Boston University School of Public Health on a new four-year federally funded FASD health services research initiative in the Military Health System (MHS). Launched with the workshop, the FASD Prevention and Clinical Guidelines Research initiative will investigate the impact of FASD on military families and the working-age population including parents and prospective parents on factors such as resource allocation, burden of disease, quality and efficiency of care, access to care, and population-level health outcomes. Project findings will support the development of medical and behavioral health interventions to support individuals with FASD and their military families. The project’s evidence base will inform clinical practice guidelines for replication in civilian systems of care.

Operated within the U.S. Department of Defense (DoD), the MHS is one of America’s largest and most complex health care institutions and the world’s preeminent military health care delivery operation. The MHS provides health services through both direct and private sector care to approximately 9.6 million beneficiaries, composed of uniformed service members, military retirees, and their family members. CHSR provides direct support services to DoD and the MHS, conducts research in support of MHS strategic goals and objectives, provides Health Services Research (HSR) training to students and faculty, and supports civilian HSR organizations. FASD United is the voice of the FASD community and strives to expand the capacity of FASD-informed diagnostic, medical, behavioral health, and non-clinical services, advocate for policy changes guaranteeing full inclusion in all systems of care and benefits programs for children and adults living with FASD, and prevent prenatal exposure to alcohol and other harmful substances. Dr. Rachel Sayko Adams from the Boston University School of Public Health is a longstanding collaborator with USUHS who is a nationally recognized expert on alcohol use (AU) and alcohol use disorders (AUD), particularly in the military population.

## Introduction

The Uniformed Services University of the Health Sciences (USUHS), in conjunction with FASD United, hosted the *Workshop on Fetal Alcohol Spectrum Disorders Prevention and Clinical Guidelines Research* on 21 September 2022 in Washington, DC. Organizers planned the workshop through weekly meetings from April to September 2022. Speakers were chosen purposefully in collaboration with USU, Boston University School of Public Health and FASD United with the goal of highlighting the work of the project, state of the science from genetics to lived experience of patients and families living with FASD, and the unique needs of military families. More than 50 attendees from academia, healthcare, federal agencies and consumer advocacy organizations gathered in-person or virtually to review the latest research and discuss key issues surrounding fetal alcohol spectrum disorders (FASD) during the day-long workshop (see Appendix for a list of attendees).

Prof. Tracey Pérez Koehlmoos, Director of the Center for Health Services Research at USUHS; Tom Donaldson, FASD United President and CEO; Kate Boyce Reeder, FASD United Board of Directors Chair; and Susan Shepard Carlson, Chair of the FASD United Legislative and Policy Committee, welcomed attendees and set the stage for the event. USUHS, funded by the Department of Defense (DoD), trains active duty service members to become healthcare professionals and support the readiness of America's warfighters. Providing the best care and improving health outcomes for the nearly 10 million military beneficiaries across the U.S. is a top USUHS priority. USUHS researchers also analyze data generated from this care to inform decisions, improve policies, and meaningfully impact patients’ lives. Given that pregnancy and delivery as well as pediatric care are common healthcare needs for military families, addressing the burden of FASD is a particularly important health issue for this community.

The workshop marks the first milestone in a four-year research program focused on FASD prevention and the recognition of FASD in the military health system. It was organized to provide an overview of the existing research, programs, and clinical practice guidelines surrounding prenatal alcohol use and FASD. Given the complexity of symptoms, the stigma that an FASD diagnosis carries, and the lack of consensus among experts on a standard diagnostic tool or clinical guidelines for diagnosis and management, the intent of this workshop was to bring together experts from diverse specialties to provide a multifaceted approach to future research. In the coming years, the program will embark on an environmental scan of the military health system and a community needs assessment to understand the lived experience of people with FASD and their caregivers. These activities will be followed by a final line of effort in which researchers will apply modeling and predictive analytics to patients’ electronic health records (EHRs) in an effort to improve early diagnosis and patient outcomes in the future. USUHS’s external partners in this prevention plan are FASD United, Boston University School of Public Health, and the Henry M. Jackson Foundation for the Advancement of Military Medicine; internal USUHS collaborators are the Center for Health Services Research (CHSR); the School of Medicine Departments of Preventive Medicine & Biostatistics, Clinical Psychology, Gynecologic Surgery and Obstetrics, Family Medicine, and Pediatrics; and the Graduate School of Nursing.

The workshop began with a series of presentations on FASD risk factors and causes, strategies for diagnosis and interventions, and impacts and lived experiences. Individuals and families affected by FASD spoke about the ways FASD, its symptoms, and the social stigma associated with it influences their daily lives, experiences at school and work, and access to healthcare. Several speakers highlighted the work of non-profit organizations and advocacy groups in supporting families affected by FASD and other challenges faced by military families more broadly. Finally, the workshop closed with a discussion of federal agency perspectives that highlighted initiatives aimed at advancing research, surveillance, public health prevention, and access to care for individuals and families affected by FASD. The following sections summarize key insights from each presentation.

### FASD risk factors and impacts

#### Jeffrey R. Wozniak, University of Minnesota

FASD is estimated to affect 2–5% of babies born each year in the U.S., including both diagnosed and undiagnosed individuals. Wozniak discussed FASD risk factors, how alcohol affects development, and the importance of early diagnosis and intervention.

#### FASD risk factors

FASD is caused by alcohol consumption during pregnancy, a factor that is common, complex, and poorly understood. Alcohol use during pregnancy is often unintentional – women may choose to drink without knowing that they are pregnant or may be unaware of the impact that small amounts of drinking can have on their baby. Further, it is not limited to individuals of any particular socioeconomic status. Known risk factors include a late awareness of pregnancy, drinking patterns before pregnancy, confusion over what constitutes “binge drinking,” and the father’s drinking habits during pregnancy [1–3]. The prevalence and prevention of FASD is a complex social problem involving factors such as a culture of drinking, unclear messaging from health professionals, sexual behavior, and the difficulties of habit change.

#### How alcohol affects development

Alcohol exposure during pregnancy can affect biochemical and physical development at any time during gestation. Interfering with fetal development at a system-wide level, alcohol can cause a wide range of birth defects and disabilities. Babies with FASD can have significant impairment to their central nervous systems, eyes, ears, hearts, and other organs and body systems. Alcohol use can also contribute to miscarriages and stillbirths, especially when combined with tobacco [4]. Research in mice in the earliest stages of pregnancy showed clear developmental effects, even after a single exposure to alcohol [5, 6]. Brain scans of children with FASD show underdevelopment in critical places like the hippocampus, the corpus callosum, cortical folds, and white matter [7, 8].

FASD presents in many different ways, from intellectual difficulties to mood dysregulation, poor executive functioning and working memory, increased naiveté, and related behavioral issues. FASD can also affect mental health; at the severe end of the spectrum, individuals have high rates of attention-deficit/hyperactivity disorder (ADHD), depression, substance abuse, and other disorders [9, 10]. However, Wozniak noted that children with FASD also have strengths, such as higher levels of sociability, determination, and resilience.

#### The importance of early diagnosis and intervention

Although prenatal alcohol exposure causes foundational issues that damage a child’s brain and body, children with FASD can often overcome these challenges with the right interventions. Early, targeted interventions tailored to a child’s specific symptoms can have a positive, compounding effect on their brains and their lives. For example, children with FASD may have difficulty orienting their attention to someone, which can affect emotion recognition and affiliation and lead to emotion dysregulation. When this symptom is recognized early on, healthcare providers can recommend specific interventions to improve emotional learning.

Early diagnosis is crucial to deriving the greatest benefit from FASD interventions and improving outcomes. Early diagnosis also raises awareness of FASD, which can prevent alcohol use during subsequent pregnancies, especially in families who are at higher-risk. Unfortunately, Wozniak noted that current diagnostic tools are inadequate, and possibly overly reliant on racially-biased facial characteristics, making early diagnosis an important ongoing challenge for the field.

### Trends and issues in at-risk alcohol use

#### Rachel Sayko Adams, Boston University, Brandeis University, and Veterans Health Administration; Joshua Gray, USUHS School of Medicine

To provide background on the alcohol use patterns that can contribute to prenatal drinking and FASD, Adams and Gray discussed at-risk alcohol use (drinking more than recommended daily or weekly limits) among women generally and in the military specifically.

#### At-risk alcohol use among women

Traditionally, women who were parents drank less than non-parents. However, several factors have contributed to a rise in alcohol consumption and in alcohol-related deaths in recent years, especially among women of child-bearing age [11, 12]. For example, some cultural messages – especially those circulating on social media – approve of or even encourage mothers to drink. Studies examining the rise of “wine mom” culture show how drinking has come to be viewed as a socially-acceptable form of self-care or empowerment [13, 14], though articles countering these messages have also appeared [15, 16]. The COVID-19 pandemic also increased alcohol consumption above pre-pandemic levels [17, 18]. Given these trends, Drs. Adams and Gray suggested that more research is needed – not to shame women, but to draw attention to and understand this important public health issue.

#### At-risk alcohol use in the military

The military community has a strong drinking culture. Binge drinking, associated with many negative health consequences, is more prevalent in both male and female service members than those in other occupations [19, 20]. In 2013, the Institute of Medicine declared substance misuse (and especially alcohol misuse) among military personnel and their dependents to be a public health crisis [21]. Studies show that female service members drink at lower rates than males but face a higher risk for drinking-related problems, especially as combat exposure increases [22, 23]. Research also shows that female service members with at-risk drinking habits frequently have other comorbidities or unhealthy behaviors, increasing their risk for substance misuse and self-harm, which impacts military readiness and requires significant interventions [24]. In addition, up to 20% of military wives report at-risk drinking behavior. Given that more than 100,000 babies are born every year to military families, it is important to learn more about military wives and military families as a whole [25, 26].

In addition to these concerning trends, another key challenge is the degree to which at-risk alcohol use is recognized and treated among military service members and their families. Many healthcare providers still find it difficult to ask patients about their alcohol use, define binge drinking, and refer people to treatment. For example, an analysis of post-deployment health screenings showed that a non-trivial proportion of service members fall into the “at-risk” or “severe risk” range for alcohol abuse [27], yet fewer than half are invited to follow-up assessments [28, 29]. Unfortunately, service members who admit to substance misuse or seek help often face stigma among their colleagues and community. Participating in treatment is known to have negative career consequences, increasing the temptation to answer screening questions dishonestly. The military is taking measures to mitigate this stigma, such as by improving patient confidentiality protections, but it remains a significant challenge.

### Screening and intervention for perinatal substance use

#### Amy Loree, Henry Ford Health

Screening for alcohol use, paired with interventions, can improve the health of women and their children. However, screenings are not always effectively deployed and do not always result in access to needed care. Loree discussed methods for preventing and identifying prenatal drinking and a new technology-based intervention to increase screening rates and effectiveness.

#### Strategies for preventing prenatal drinking

In a 2016 analysis, roughly 10% of pregnant people reported alcohol use within the past month, and about 40% of those individuals also reported other substance use. Yet, only 10% of all individuals who need treatment receive care [30]. In addition, inconsistent messages about drinking during pregnancy can be confusing and minimize the perceived threat.

To prevent prenatal drinking, public health messaging now focuses on *all* women of childbearing age who may be unhealthy drinkers. This strategy has been adopted for two reasons. First, preconception drinking is a strong risk factor for prenatal drinking. Second, half of all pregnancies are unintended, which means that many women do not realize they are pregnant until after the critical early development period. Interventions for at-risk drinking behavior also include contraception plans for those who don’t want to get pregnant.

#### Electronic screening at scale

To identify and reduce prenatal drinking, healthcare providers use a process called screening and brief intervention (SBI), which has been shown to reduce substance use [31]. With SBI, every adult is screened for substance use during routine care, and the interventions are tailored to their responses. For example, if a person is planning a pregnancy, the intervention would advise alcohol cessation. If the person needs help to stop drinking, the intervention would refer her to additional services or treatment and frequent follow ups.

Unfortunately, while SBI is intended to be brief, including it in appointments is often unrealistic given the lengthy list of topics clinicians are responsible for screening and counseling on. To address this barrier, Loree and her team created a customizable electronic SBI (e-SBI) platform that people can use to fill out a screening questionnaire before their appointment. Studies found that the e-SBI increased provider fidelity and respondent disclosure of sensitive information; reduced costs, training requirements, and provider time; demonstrated a strong association between abstinence from substances and healthy birth outcomes; and was well-accepted by patients [32–35].

At-scale implementation of this e-SBI is now underway at Henry Ford Health, a large, integrated health system that serves both Detroit and rural areas with a diverse patient population. The e-SBI asks women ages 18–45 about a range of issues, including alcohol use, and integrates into EHRs, a key detail to help providers understand patient behavior and needs. It requires minimal staff training, includes best practice advisories, and is easy to set up and use. Despite a busy clinical environment, COVID-19 disruptions, and the already-high frequency of patient messages, Loree expressed confidence that implementation will ultimately be successful and sustainable.

### Overcoming stigma and discrimination to prevent FASD

#### Kathleen Mitchell, FASD United

Mitchell, who has a daughter with fetal alcohol syndrome, shared her own story to inspire women to seek help, encourage healthcare providers to speak with their patients about substance use, and galvanize researchers to continue to study FASD prevention and interventions [36]. She discussed the growing challenge of perinatal substance use; how stigma, bias, and discrimination influence FASD prevention and care; and how families affected by FASD can thrive when they are given the treatment, empathy, and support they need.

#### The growing challenge of prenatal substance use

There is no known safe amount of prenatal alcohol exposure. Prenatal drinking can lead to vastly different outcomes in children, and researchers do not fully understand the factors that may increase vulnerability to alcohol exposure or how alcohol exposure interacts with other factors that affect fetal and child development. Yet despite the dangers, prenatal alcohol use is increasing. Binge drinking while pregnant is especially dangerous, and it is often associated with the use of other substances, such as tobacco or marijuana. As marijuana use has become legal in an increasing number of states, prenatal marijuana use has also risen. Cultural messages describe it as safe to use during pregnancy, but research has shown that marijuana can amplify alcohol’s teratogenic effects (causing birth defects or fetal abnormalities) in animals, suggesting this combination potentially could increase the severity of FASD [37]. To address these issues and combat misleading messages, Mitchell said that it will be important to make women of childbearing age aware of the hazards of prenatal drinking and substance use.

#### Removing stigma and providing support

In addition to building awareness, it is crucial to support the avoidance of prenatal substance use. Many women who drink prenatally cannot stop on their own, even if they want to. Addiction is a treatable disorder, but the stigma around alcohol use disorder and FASD leaves many women embarrassed to admit they drink, ashamed to ask for help, and worried that they will lose custody of their children. Women need to feel safe – not stigmatized – when discussing prenatal drinking with their healthcare provider [38, 39]. The stigma also leads to policy discrimination and poor resource allocation, when what is needed is better treatment and care [40].

To address this barrier, Mitchell said that healthcare providers must push past any awkwardness they feel asking patients about drinking behavior, educate themselves about available resources, and believe that mothers with addictions can – and want to be helped [41]. Addiction recovery is a difficult process and women need empathy and treatment, not judgment and discrimination.

### FASD diagnostic guidelines and criteria

#### Michelle Kuhn, University of Washington and Seattle Children’s Research Institute

Kuhn discussed FASD diagnostic criteria and shared results from her examination of FASD diagnostic systems worldwide.

#### FASD diagnostic criteria

To diagnose FASD, clinicians look for several criteria: multiple, interacting, functional central nervous system differences; prenatal alcohol exposure (PAE); sentinel facial features; and growth deficits. The broad range of criteria can make FASD difficult to diagnose, understand, and treat. Yet diagnoses are essential to determining a symptom’s cause, understanding its nature and specificity, guiding treatment, and improving people’s lives. Therefore, the criteria must be broad enough to both reflect the heterogeneity of FASD and accurately identify it so that those affected can receive appropriately-tailored interventions and support [42].

#### Existing FASD diagnostic systems

In her research, Kuhn compared ten different FASD diagnostic systems across the world, and overall found more similarities than differences. Most were built with multidisciplinary evaluation teams looking at the full FASD range of symptoms, and have a deliberately wide spectrum of diagnostic outcomes to accommodate the heterogeneity of FASD. There are slight differences in how some terms are defined, such as “binge drinking” or “consistent exposure,” as well as some differences in diagnostic requirements, such as the presence of sentinel facial features or the (self-reported) PAE threshold.

Most diagnostic systems do not require confirmed PAE in the presence of all three sentinel facial features (a thin upper lip, a smooth philtrum, and a narrow inner eye opening) which has a specificity to PAE of more than 95% [43]. The growth deficit criteria include pre- or postnatal small size, significant and consistent growth delays, and functional deficits that can be developmental, behavioral, structural, or neurological. While the diagnostic systems define functional deficits differently, overall the systems are consistent in that they require symptoms to present meaningful impairment.

### Lived experiences, strengths, and interventions for people with FASD

#### Christie Petrenko, University of Rochester and Mt. Hope Family Center

Petrenko discussed the stigma experienced by many families affected by FASD, along with new opportunities for interventions to improve the outlook for children and families. She emphasized that people with FASD have important strengths and capabilities that are often overlooked [44]. Like all of us, they need the right opportunities, relationships, and support to pursue meaningful lives.

#### The stigma of FASD

Many families with FASD face severe and pervasive stigma. This has serious consequences, especially when it leads to delayed diagnosis, under diagnosis, and discrimination in healthcare delivery, because it means that people with FASD frequently do not get the treatment, accommodations, and support they need to thrive [45, 46]. Even with an appropriate diagnosis, it can be hard for families to find FASD-informed services for a person’s particular symptoms, which can be easily misunderstood and misattributed, and can contribute to a stressful home and family life [47].

For mothers and children with FASD, this stigma also leads to feelings of shame and guilt. Individuals with FASD report feeling different, unsupported, and discriminated against [48]. Despite the many difficulties, caregivers work hard to support, protect, and advocate for their children; access treatment; educate themselves; and minimize future problems [49]. Unfortunately, these added burdens can increase feelings of stress and burnout.

#### New opportunities for interventions

Research shows that people with FASD and their families do benefit from traditional “deficit-focused” interventions. However, going beyond this to embrace person-centered, strengths-based interventions can help people not just survive but thrive, by helping them to feel valued, supported, and included [50–54]. New strategies – at the individual, policy, and systems level – could improve the accessibility of such interventions for families and providers; broaden the age ranges served to include early childhood, adolescence, and adulthood; address mental health needs; and expand outcomes to focus on success, not merely deficit reduction.

Petrenko shared three projects that aim to improve the lives of people with FASD. First, her team is testing a self-directed mobile app that assists healthcare providers by offering learning modules, videos, and support for families. Another project, in partnership with the Adult Leadership Collaborative on FASD, is creating a different mobile app for adults with FASD, aimed at addressing the core psychological needs – autonomy, competence, and connection – that impact physical health and quality of life [55]. While these two app-based projects are promising, Petrenko offered the caveat that smartphone and internet-based interventions are not accessible to everyone. In a third project, her team is creating a tool, based on Extension of Community Healthcare Outcomes (ECHO) approaches, to train providers to recognize FASD, increase diagnoses, and, ideally, point families with FASD to new strengths-based interventions.

### Panel discussion: individuals and family members living with FASD

#### Jenn Wisdahl, Moderator, Rebecca Tillou, Sean Bousquet, and Laura Bousquet, Panelists

Recognizing patience, resilience, forgiveness, and the many other strengths of people with FASD can help remove the stigma surrounding it and enable others to see people with FASD as whole people, not outcasts. To that end, Wisdahl, herself the parent of three teens with FASD, invited Rebecca Tillou, Sean Bousquet, and Sean’s mother Laura Bousquet, to share their stories with workshop participants and offer their advice to make the world an easier, better, more understanding place for people living with FASD.

Panelists began by sharing their personal experiences with FASD. Bousquet noted that he was not diagnosed with FASD until his teenage years, though he experiences many symptoms that affect his daily life. These include cognitive functioning issues, an inability to read emotions, heart murmurs, exotropia, hearing impairment, sleep disorders, and bone deformities. As a younger child, he was given many other diagnoses, including oppositional defiant disorder, trauma, autism, and severe ADHD. Frequent changes in school and home life also created challenges, but once those stabilized he was able to become a passionate advocate for people with FASD.

Tillou, 42, was not diagnosed until adulthood. As a child, she was very small and frequently sick, but she thrived in loving, structured home and school environments. In her thirties, she experienced problems with executive functioning, and at the same time learned that her birth mother had been an alcoholic. Although it was difficult, she was able to get diagnosed with FASD as an adult, and has also become an FASD advocate.

Wisdahl invited panelists to share their thoughts about how society can better support people living with FASD. Laura Bousquet suggested that every child could be given a teratogen test at birth, because having a diagnosis as soon as possible would help affected people access critical early interventions. In addition, a public education campaign for community leaders, school administrators, and law enforcement could promote a wider understanding of FASD and prevent interpersonal problems. Tillou agreed, adding that parents could also benefit from such an education campaign, and also stressed that all people must learn to open their hearts to those who are struggling, whether or not they have an FASD diagnosis.

Laura Bousquet added that lawmakers should be made aware of the importance of giving children with FASD access to special services. FASD is a real neurodevelopmental disorder that affects millions of people. In his experience, Sean Bousquet said that some special education teachers view FASD as an excuse, not a disorder, and set unreasonable expectations for school performance despite being told explicitly what the challenge was.

Asked what advice to give to a new self-advocate, Tillou suggested learning as much about FASD as possible, finding FASD resources online, and telling your own story widely, even if people try to silence you. Sean Bousquet noted that carrying business cards explaining his FASD symptoms has been an effective way to share his story and help others understand his condition.

### FASD United Affiliate Network

#### Susan Elsworth, FASD United and the Indiana Alliance on Prenatal Substance Exposure

Elsworth, the parent of multiple children with FASD, described the mission and impacts of the FASD United Affiliate Network, a large, diverse group of organizations collaborating to reduce PAE, improve the lives of families affected by PAE, and advocate for systemic change in PAE and FASD research.

The 37 organizations of the FASD United Affiliate Network operate autonomously across 28 states, creating programs and interventions that align with their strengths, demonstrate their values, and bring hope to those in need. They may sponsor research, offer training or interventions, support families, or advocate for PAE prevention. For example, the network has driven a groundswell of support for Congressional action on the FASD Respect Act, legislation that would authorize federally-supported programs for state, tribal, and private entities to collaborate on PAE prevention, screening, identification, research, and FASD-informed services. Some organizations within the network are run by volunteers or a small staff and operate on a very small budget; others are much larger. The network also benefits from an international collaborative that contributes experience and knowledge.

The FASD United Affiliate Network has enhanced FASD awareness at multiple civic levels, increased mandated FASD training and screenings, improved diagnostic capacity, and even created FASD housing. In addition, members have shared their stories as widely as possible, helping to remove the shame associated with FASD via a national anti-stigma campaign; turned research into accessible, practical ideas for families, schools, and healthcare providers; incorporated diversity initiatives to reach and represent more people; and brainstormed ideas for engaging community partners.

As one specific example of this work, Elsworth described a state-wide needs assessment the organization performed in Indiana. The effort sought to learn what individuals, caregivers, and service providers across the state already know about FASD and FASD services and then craft a narrative to explain to state legislators why more FASD services and research are needed. In response to a question, Elsworth noted that to connect with military families, the FASD United Affiliate Network will seek to create a partnership with Exceptional Families of the Military, whose existing programs to assign service members to locations that meet the health and educational needs of their families align perfectly with the FASD United Affiliate Network’s mission.

### Exceptional Families of the Military

#### Austin Carrigg, Exceptional Families of the Military

Carrigg described the work of Exceptional Families of the Military (EFM), a non-profit organization that supports military family members living with disabilities. EFM’s all-volunteer staff understand both the stresses and disruptions of military life and the needs and experiences of families with a disability, and the organization helps these families in three ways. First, it organizes peer-to-peer support groups for healthcare providers, adults with disabilities, and people with specific healthcare needs. More than 9,000 families currently participate in these groups, with new people joining every day. Second, EFM supports case management for one-on-one consulting to identify and meet a family’s civilian and military needs. For example, services can include helping families to find new healthcare providers or complete compassionate reassignment requests. Finally, the organization advocates for policy and legislation reform to raise awareness of issues facing military families and encourage Congress to fix service gaps in order to better reflect the lived experiences of these families.

Frequent reassignments are a particularly challenging part of military life, because they mean that every few years, families must find new healthcare providers, who often have long wait lists. To address this, EFM created the EFM Binder to help families find the services they need whenever they are relocated, along with a Permanent Change of Station checklist to help military families coordinate the care and services they need. In addition, EFM social media campaigns help to raise awareness of the organization and its mission, identify those in need, and connect families across long distances. Finally, the EFM Network Partner Program vets organizations to ensure their safety and integrity.

EFM also works in tandem with a variety of other organizations including Tricare for Kids, the White Oak Collaborative, the EFMP Coalition, and the Hidden Helpers Coalition. With these partners, EFM works to identify and fix gaps in care, such as the multiple specific qualifying criteria of the Tricare Extended Care Health Option (ECHO), which was established by Congress to eliminate the years-long waitlists for location-based Medicaid waivers but is itself difficult to navigate. Tricare ECHO also conducts studies evaluating disability therapies, such as for autism, and EFM helps military families enroll in them.

### Blue Star Families

#### Jessica Strong, Blue Star Families

Strong discussed the work of Blue Star Families and detailed the results of its annual Military Family Lifestyle Survey [56]. Blue Star Families was founded in 2009 by military spouses who wanted to improve the lives of families of service members and veterans. That year, 2,700 military-affiliated families responded to a national survey that sought to create a holistic picture of their challenges and needs. The survey is now conducted annually, and the results are used to identify research ideas, provider recommendations, new partnerships, and specific actions and resources to meet those needs.

For the 2021 survey, Blue Star Families collected responses from 8,000 people including service members on active duty, in the National Guard, or Reserves; their spouses; and veterans. The most common challenge respondents identified was the physical separation from family members. This separation can be because of active-duty requirements or because parents choose to live separately to spare children the disruption of moving (which often entails enrolling in new schools, navigating long wait lists for healthcare providers, and adjusting to a lack of nearby friends or family). The second challenge identified was the high unemployment rate among military spouses – over four times the national average – which contributes to financial stress and commonly results from frequent moves or challenges with childcare, especially if children have special needs like FASD.

While most children in military families are thriving, they are affected by their own set of challenges. Just over half of respondents noted that they had a child with one or more special needs, such as FASD, ADHD, or a learning disability. While about 17% of respondents reported that their children receive mental healthcare, another 17% reported that they *wanted* their child to receive mental healthcare but were wary of telehealth, couldn’t find an available provider or convenient appointment time, or were concerned about a mental health diagnosis jeopardizing a child’s future military career. This last concern represents an important barrier because children in military families join the military in greater numbers, and, unlike children in civilian families, the military has access to their lifetime medical records.

### Panel discussion on the federal response to FASD

#### Bill Dunty and Tatiana Balachova, National Institute on Alcohol Abuse and Alcoholism (NIAAA); Dawn Levinson, Health Resources and Services Administration (HRSA); Elizabeth Dang, Centers for Disease Control and Prevention (CDC); and Sharon Newburg-Rinn, Administration for Children & Families (ACF) in the U.S. Department Health & Human Services (HHS)

Tom Donaldson introduced the speakers for the workshop’s final session, a panel discussion of the federal agency perspective and the federal response to FASD. He noted that federal agencies have helped discover much of what is now known about FASD through funding, expertise, and leadership. Panelists described the FASD work their agencies conduct and participated in a brief open discussion with workshop attendees.

#### Bill Dunty, NIAAA

Dunty is the FASD research coordinator at the NIAAA, the world’s largest funder of alcohol research. Established in 1970, the NIAAA shares its findings with the public and with healthcare providers to improve prevention, diagnosis, and treatment of alcohol-related problems at all stages of life. FASD-specific funding opportunities represent about 7% of the NIAAA’s budget and cover PAE prevention and FASD causes, diagnoses, and interventions. For example, a study done in collaboration with several other federal agencies and women’s health centers found that combined prenatal alcohol and tobacco exposure increases the risk of sudden infant death syndrome (SIDS) and stillbirth [4, 57]. Another study, an ongoing longitudinal observation of 7,500 pregnant women and children, aims to establish brain development trajectories of children with and without prenatal substance exposure.

The NIAAA also funds research on PAE identification and its impact on chronic disease, the impact of fetal co-exposures on the placenta and gut biome, intervention development and implementation, and reducing the FASD stigma. In addition, his team is in the process of harmonizing the global FASD classification systems that Kuhn described to enable consistent global diagnoses, prevalence studies, and research findings. Finally, the Institute’s Healthcare Professionals’ Core Resource on Alcohol, launched recently, was developed to help busy clinicians gain a better understanding of how alcohol impacts health and find resources for screening and intervention.

#### Tatiana Balachova, NIAAA

Balachova serves as the Executive Secretary of NIAAA’s Interagency Coordinating Committee on Fetal Alcohol Spectrum Disorders (ICCFASD), whose mission is to improve coordination, cooperation, and collaborations surrounding FASD with federal agencies such as the CDC, ACF, and HSRA. The ICCFASD’s work includes research to improve clinical services, quality of life, education, monitoring, and prevention. Working groups tackle specific topics and identify opportunities for collaboration, and annual public meetings share research findings and expertise. At the most recent meeting, for example, attendees learned about the lived experiences of adults with FASD from the FASD Adult Leadership Committee, especially regarding the need for FASD-informed healthcare and mental health providers.

#### Dawn Levinson, HRSA

Levinson works in HRSA’s Maternal and Child Health Bureau (MCHB), whose mission is to improve the health, mental health, and well-being of the nation’s mothers, children, and families through improving access to and equity of services and support across their lifespan; strengthening public, mental, and behavioral health capacities; encouraging culturally sensitive, trauma-informed, and community-based services; and maximizing impact through performance tracking and leadership, partnership, and stewardship initiatives. For example, the MCHB led the creation of the National Maternal Mental Health Hotline, through which women receive free, real-time, confidential, professional, sensitive, evidence-based emotional and logistical support for mental health or substance use needs in English or Spanish. Through grants, the MCHB also sponsors projects like the SAFEST Choice Learning Collaborative, a primary care provider education program to reduce PAE and improve FASD outcomes through an extensive, skills-based training curriculum in areas within states, territories, and tribal nations that experience high rates of prenatal drinking. Preliminary results demonstrate increased healthcare provider knowledge of FASD and improvements in clinicians’ screening ability, knowledge of community resources, and clinical practices.

#### Elizabeth Dang, CDC

Dang, of the CDC’s National Center on Birth Defects and Developmental Disabilities, works on FASD-related projects such as monitoring alcohol use during pregnancy, sharing and implementing evidence-based PAE interventions, and public education around PAE and FASD. The target audience for these projects includes the 1 in 7 pregnant people who report some drinking, the 1 in 20 who report binge drinking, and healthcare providers [58]. For example, the CDC is currently revamping its extensive resource library to improve provider education and communication around FASD, and has also recently awarded several large, multi-year grants for FASD research.

The CDC frequently collaborates on FASD projects. For example, cooperative agreements to implement alcohol prevention SBIs at several large women’s health systems across the U.S. have screened more than 100,000 people to date. To reach healthcare professionals, the CDC collaborates with national professional organizations, such as a longstanding FASD-focused partnership with the American Academy of Pediatrics to improve awareness, diagnosis, interventions, health, and development outcomes of children affected by PAE. In addition, CDC and FASD United have a cooperative agreement to raise awareness of the risks of PAE, improve the lives of people with FASD, and promote FASD prevention. Finally, CDC and ACF work together to improve the identification and care of children with PAE within the child welfare system.

#### Sharon Newburg-Rinn, ACF

ACF collects data about children in the welfare system, in foster care, or otherwise receiving care from HHS. Through interviews, ACF discovered that FASD is underdiagnosed because, in addition to a lack of interagency coordination, few professionals involved were even asking about PAE and many subscribed to the widely-held belief that alcohol use during pregnancy is not a serious issue. To counter this problem, ACF is collaborating with several other organizations to launch a large project to improve identification and care of children with PAE, including through a nationally-accessible FASD toolkit for child welfare professionals and caretakers.

## Discussion

In a brief open discussion, participants explored misinformation, collaboration, and potential relationships between autism spectrum disorder and FASD.

Newburg-Rinn expanded on the widespread misinformation around PAE and FASD. Many staff members at child welfare agencies looked only for the sentinel facial features, and were unaware that FASD can be present without these. Balachova pointed out that research shows that children with the sentinel facial features have better outcomes because they are more likely to be diagnosed early and receive services. A participant agreed, noting that her own child was initially misdiagnosed because she lacked the sentinel facial features. This underscores the importance of training healthcare and welfare professionals to properly identify FASD through the full range of its diagnostic criteria.

Susan Carlson asked if the ICCFASD still collaborated with the Department of Education and the Department of Justice’s Office of Juvenile Justice and Delinquency Prevention, both of which could earmark funding for FASD research. Balachova replied that due to staff changes, those collaborations have been paused, but the ICCFASD would like to restart that relationship, which included creating FASD-informed materials for legal and educational professionals, and expand the community around improved FASD services.

A participant asked if the co-occurrence of FASD and autism was under investigation. Kuhn replied that her clinic is located within an autism center, creating an opportunity for collaborative research to tease apart the potential similarities, differences, and co-existence rates for these two conditions. Balachova added that individuals with FASD are overall far more likely to have other physical or developmental disorders and poor health outcomes, making it imperative to learn more about how FASD affects a person’s entire lifespan.

### Closing remarks

#### Tracey Pérez Koehlmoos, USUHS, and Tom Donaldson, FASD United

Koehlmoos and Donaldson thanked attendees and speakers for participating in the workshop and expressed gratitude to the broader FASD community for sharing their attention, inspiration, and expertise. Closing the workshop on an optimistic note, they said it will be exciting to see how the research projects and advocacy programs described during the event move the community closer to its goal of improving the services for – and the lives of – children, adults, and families affected by FASD.

### Future Research

Following this workshop, the first phase of the four-year research project will begin with an environmental scan as referenced earlier. Current knowledge of prevention, diagnosis, and management of FASD presented in the workshop will help shape the scan in terms of what materials and resources should be sought within the MHS. Additionally, the insights shared by the individuals and families living with FASD will help guide the needs assessment that will include MHS providers and military families living with FASD. Culmination of this research project will lead to actionable items and improved care delivery for military families and serve as a model for healthcare systems throughout the private sector.

## Appendix 

**Table 1:** Workshop Attendees

Organization Type	Organization	Participant
GovernmentCOL	Centers for Disease Control and Prevention	Elizabeth Parra Dang, MPH
	Health Resources and Services Administration	Dawn Levinson, MSW
	James Bell Associates (HHS Contractor)	Crystal Coles, PhD
		Erin Ingoldsby, PhD
	HHS, Children’s Bureau, Administration for Children & Families (ACF)	Sharon Newburg-Rinn
	Madigan Army Medical Center	LTC Jacob Hogue, MD
		LTC Bonnie Jordan, MD
		Tyler Raymond, DO, MPH
		COL Jason Pates, MD
	National Institute on Alcohol Abuse and Alcoholism	Tatiana Balachova, PhD
		Bill Dunty, PhD
	Uniformed Services University of the Health Sciences	Binny Chokshi, MD, MEd
		Paul Crawford, MD
		Joshua Gray, PhD
		Dean Lynette Hamlin, PhD, CNM
		COL Patrick Hickey, USA, MD, MPH
		Beth Hisle-Gorman, PhD
		Tracey Koehlmoos, PhD, MHA, Co-PI
		Elizabeth Lee, DrPH, MPH, Co-PI
		CDR Monica Lutgendorf, USN
		COL Dana Nguyen, USA, MD
		COL Cadet Nyland, USAF, MD
		Rosemary Payne, MSN, BSN, RN
		LtCol, Jeanmarie Rey, USAF, MD
		Jennifer Trautmann, PhD, RN, FNP-BC
Non-profits	Blue Star Families	Jessica Strong
	Exceptional Families of the Military	Austin Carrigg
	FASD United	Kate Boyce Reeder
		Susan Carlson
		Tom Donaldson
		Susan Elsworth
		Heather French
		Kathleen Mitchell, MHS, LCADC
		Jenn Wisdahl
Patients and Families		Laura Bosquet
		Sean Bosquet
		Rebecca Tillou
Academic research organizations	Boston University	Rachel Sayko Adams, PhD, MPH
	Henry Ford Health Center for Health Policy & Health Services Research	Amy Loree, PhD
	Henry M. Jackson Center for the Advancement of Military Medicine, supporting the Center for Health Services Research at USUHS	Amanda Banaag, MPH
		Miranda Janvrin, MPH
		Luke Juman, MPH
		Cathaleen Madsen, PhD
		Shatonya Lumpkin
	University of Minnesota Medical School	Jeffrey Wozniak, PhD, LP
	University of Rochester	Christie Petrenko, PhD
	University of Washington	Michelle Kuhn, PhD

## Data Availability

All data is contained within the workshop report.

## Abbreviations

FASD: Fetal alcohol spectrum disorders
ADHD: Attention-deficit/hyperactivity disorder
USUHS: Uniformed Services University of the Health Sciences
CHSR: Center for Health Services Research
MHS: Military Health System
DoD: Department of Defense
HSR: Health Services Research
AU: Alcohol use
AUD: Alcohol use disorder
HER: Electronic health record
SBI: Screening and brief intervention
e-SBI: Electronic screening and brief intervention
PAE: Prenatal alcohol exposure
ECHO: Extension of Community Healthcare Outcomes
EFM: Exceptional Families of the Military
ECHO: (TRICARE) Extended Care Health Option
NIAA: National Institute on Alcohol Abuse and Alcoholism
HRSA: Health Resources and Services Administration
CDC: Disease Control and Prevention
ACF: Administration for Children & Families
HHS: U.S. Department Health & Human Services
SIDS: Sudden infant death syndrome
ICCFASD: Interagency Coordinating Committee on Fetal Alcohol Spectrum Disorders
MHCB: Maternal and Child Health Bureau
